# The Expanding Role of Vesicles Containing Aquaporins

**DOI:** 10.3390/cells7100179

**Published:** 2018-10-22

**Authors:** M Carmen Martinez-Ballesta, Paula Garcia-Ibañez, Lucía Yepes-Molina, Juan José Rios, Micaela Carvajal

**Affiliations:** Group of Aquaporins, Plant Nutrition Department, Centro de Edafologia y Biologia Aplicada del Segura, CEBAS-CSIC, Campus Universitario de Espinardo, E-30100 Murcia, Spain; mballesta@cebas.csic.es (M.C.M.-B.); pgibanez@cebas.csic.es (P.G.-I.); lyepes@cebas.csic.es (L.Y.-M.); jjrios@cebas.csic.es (J.J.R.)

**Keywords:** aquaporins, membrane vesicles, trafficking, cell communication, biofilters

## Abstract

In animals and plants, membrane vesicles containing proteins have been defined as key for biological systems involving different processes such as trafficking or intercellular communication. Docking and fusion of vesicles to the plasma membrane occur in living cells in response to different stimuli, such as environmental changes or hormones, and therefore play an important role in cell homeostasis as vehicles for certain proteins or other substances. Because aquaporins enhance the water permeability of membranes, their role as proteins immersed in vesicles formed of natural membranes is a recent topic of study. They regulate numerous physiological processes and could hence serve new biotechnological purposes. Thus, in this review, we have explored the physiological implications of the trafficking of aquaporins, the mechanisms that control their transit, and the proteins that coregulate the migration. In addition, the importance of exosomes containing aquaporins in the cell-to-cell communication processes in animals and plants have been analyzed, together with their potential uses in biomedicine or biotechnology. The properties of aquaporins make them suitable for use as biomarkers of different aquaporin-related diseases when they are included in exosomes. Finally, the fact that these proteins could be immersed in biomimetic membranes opens future perspectives for new biotechnological applications.

## 1. Introduction

Intercellular communication in animals and plants is a fundamental biological process in which extracellular vesicles, including exosomes, have a determinant role. In the fields of biology and medicine, interest in vesicles as intracellular communication elements has recently increased. Among the proteins present in membrane vesicles, aquaporins (AQPs) are tetrameric intrinsic membrane proteins that facilitate the transport of water and small solutes across biological membranes [1]. The AQP monomer has six membrane-spanning helical domains and two helical segments forming a narrow aqueous pore. Two conserved motifs (asparagine–proline–alanine, NPA motifs) are localized in the narrow central constriction of the channel, playing a crucial role in water permeation and solute selectivity [1,2].

AQPs are present in almost all living organisms, including eukaryotes and prokaryotes. However, while there are 13 types of AQPs in mammals [3], in plant species, the number of isoforms is higher, for instance, 35 in *Arabidopsis thaliana*, 55 in *Populus trichocarpa*, 66 in Glycine max, and 71 in *Gossypium hirsutum* [4,5,6,7,8].

Four AQPs subfamilies have been identified in animals: water-specific channels (AQP0, 1, 2, 4, 5, 6), aquaglyceroporins (AQP3, 7, 9, 10), water and ammonium aquaporins (AQP8), and unorthodox aquaporins (AQP11, 12) [9]. In higher plants, seven subfamilies have been described: plasma membrane intrinsic proteins (PIPs), tonoplast intrinsic proteins (TIPs), nodulin 26-like intrinsic proteins (NIPs), small basic intrinsic proteins (SIPs), uncategorized X intrinsic proteins (XIPs), hybrid intrinsic proteins (HIPs), and GlpF-like intrinsic proteins (GIPs) [8]. 

In animals, modifications or alterations in the configuration of AQPs have been related to an elevated number of diseases, such as hereditary nephrogenic diabetes insipidus, congenital cataracts, and the inability to concentrate solutes in urine [10,11]. In plants, they have an important role in regulating growth and development under stressful and nonstressful conditions through the maintenance of the plant hydraulic status [8].

In spite of the differences between AQPs belonging to these two kingdoms, a common ancient ancestor for all AQPs has been described [12]. In addition, the basic function of AQPs was conserved during the evolution from bacteria to other organisms in order to preserve the physiological processes in which they are involved. AQPs from animals and plants also share common regulatory mechanisms [13]. Therefore, the crosstalk between AQPs studies in both kingdoms may provide new perspectives in which differences and similarities could be relevant. Furthermore, as AQPs are able to enhance the water permeability of membranes with a determinant role in their stability [14], new insights into their functions can be achieved by studying their effects when incorporated into vesicles. For this, taking into account their role in the regulation of physiological processes, the possibility of using these proteins for biotechnological purposes need to be examined. 

Therefore, this review incorporates the current visions of aquaporin trafficking among membranes, their importance in the communication between cells, and their potential uses in biomedicine or biotechnology derived from their properties, including their inclusion in biomimetic membranes. The limited progress in this field is reviewed with the aim of highlighting the significant gaps that remain in our understanding.

## 2. Aquaporins Activity in Vesicles

The activity of AQPs present in vesicles and the integrity of the vesicles are determined from the osmotic water permeability (P_f_); the fast-kinetics changes in the volume of the vesicles are measured after the rapid imposition of an osmotic/solute gradient (for review see Madeira et al. [15]). Different methodologies have been proposed to measure P_f_ [16]. The light scattering method provides a semiquantitative index of cell volume, and it has been widely applied in P_f_ measurements with cells or vesicles, such as erythrocytes, suspensions of membrane vesicles and liposomes reconstituted with AQPs, or lung alveolar epithelial cells [17,18]. Other methods used to determine changes in cell volume are two-dimensional (2D) images analysis, scanning probe microscopy (SPM), atomic force microscopy (AFM), and scanning electrochemical microscopy (SECM) [16,19]. However, the development of methodology for the exact measurement of P_f_ continues to be a challenge. One of the critical points is the time needed for the establishment of a suitable osmotic gradient, and this parameter is normally underestimated during measurements. In addition, cells with high water permeability require methods with rapid gradient formation [16]. These limitations are important issues that need to be solved for studies of AQPs modulators that may have applications in therapeutics, where artifacts contributing to inaccurate estimation of cell membrane water permeability may prevent optimal results from being obtained [3].

The activity of some AQPs and their water permeation function remain controversial; this arises not only from the water permeability measurement but also the system used for protein reconstitution. Thus, it has been reported that AQP11 reconstituted in proteoliposomes exhibited normal water permeability when this was determined using a stopped-flow methodology [20]. However, Gorelick et al. [21] showed that AQP11 expressed in *Xenopus* oocytes had no water permeability. Morishita et al. [22] explained that this lack of water permeability is due to the fact that AQP11 is not targeted to the plasma membrane (PM) and stays in the intracellular organelles of the *Xenopus* oocytes. Yakata et al. [23] studied the water permeability of AQP11 (excluding detergent effects) using vesicles formed directly from Sf9 cell membranes in which AQP11 molecules are expressed. In this case, the water permeability of AQP11 was 8 times lower than that of AQP1.

## 3. Aquaporins Trafficking

Protein trafficking in the plasma membrane includes different processes, such as (1) exocytosis or secretion, in which proteins migrate to the PM or outside the cell, (2) endocytosis, in which proteins are integrated into the PM for recycling or regulation of their activity, and (3) transcytosis, which involves the transit of proteins from one specific position in the PM to another. All these processes involve the formation of membrane vesicles containing proteins. One of the most documented types of protein trafficking at the PM level is caused by the need to maintain cellular homeostasis in the face of physiological or external stimuli [13]. Therefore, regulation of the abundance and distribution of proteins in the PM is related to the interchange of water and solutes on both sides of the membrane. However, the primary requirement of plants and animals is to regulate the availability of water to their cells in order to cope with fluctuations and maintain water balance. As the cell water permeability is directly proportional to the amount of aquaporin proteins in the membranes and as these proteins are finely regulated, their translocation in vesicles is one of the most important mechanisms of control [24].

### 3.1. Protein–Protein Interaction

The protein–AQPs interaction has been described as a post-translational mechanism to control AQPs trafficking in cells. In animals, AQPs in the kidney have been frequently studied due to their involvement in one of the main functions of this organ: the reabsorption of water from primary urine. Thus, trafficking of water channel aquaporin 2 (AQP2) to the apical membrane is a key factor that regulates water reabsorption in the renal collecting ducts to maintain body water homeostasis [25]. Different proteins have been described as controllers of AQP2 trafficking; the binding target is mainly localized in the AQP2 C-terminus [26]. Some of these proteins control the cAMP signaling, such as A-kinase anchoring proteins (AKAPs) and phosphodiestereases (PDEs). It has been reported that these proteins facilitate the interaction of AQP2 with protein kinase A (PKA) and therefore aquaporin phosphorylation, which is crucial for AQPs translocation. In addition, cytoskeletal components, such as actin, play an important role in the spatial and temporal regulation of AQP2 trafficking, allowing vesicle redistribution to the PM [27,28]. 

Lysosomal trafficking regulator-interacting protein LIP5 has also been reported to be involved in AQP2 sorting to multivesicular bodies [29]; LIP5 interacted in vitro with the proximal carboxy-terminal tail (L230–D243) of AQP2, but this did not occur with AQP3 or AQP4. Other proteins included in the trafficking machinery of all AQPs in general (including those of plants) were clathrin heavy chain [30] and heat shock cognate protein 70 (Hsc70) [31,32].

It has been shown that when protein–AQPs interactions occur in a phosphorylation-dependent way, an alteration of the affinities of the protein takes place. In fact, AQP5 and AQP2 trafficking has been reported to be controlled by PKA phosphorylation at Ser156 and Thr259 in AQP5 and at Ser256 in AQP2 [33]. As phosphorylation of Ser256 in AQP2 is involved in its affinity to LIP5, a similar role has been proposed for AQP5 phosphorylation [34]. In addition, this LIP5–AQP2 interaction could also be independent of the state of Ser256 phosphorylation or Lys270 ubiquitination.

The SNAREs (soluble N-ethylmaleimide-sensitive factor protein attachment protein receptors) are a family of proteins that mediate vesicle fusion in membranes and AQPs trafficking in both animals and plants. In animals, SNARE proteins have been shown to be involved in AQP2 trafficking and were detected in the cells of the principal collecting duct, together with AQP2 vesicles [35]. However, in maize plants, the trafficking of aquaporin ZmPIP2;5 has been shown to require the presence of SNARE ZmSYP121, with a reported direct physical interaction between the two proteins that affects membrane permeability to water [36]. A similar interaction has been demonstrated in *Arabidopsis thaliana* plants between the aquaporin AtPIP2;7 and SNARE AtSYP121 and between aquaporin AtPIP2;5b and SNARE AtSYP61 [37]. In fact, Hachez et al. [37] proposed two independent pathways for PIP aquaporins that might operate in the protein migration from the trans-Golgi network (TGN) to the PM: one with the exclusive involvement of SNARE SYP121 or SYP61 alone and the other with the participation of the SYP121/SYP61 SNARE complex.

In plants, the hetero-oligomerization of AQPs has been related to their trafficking [38,39]. In this way, in maize, the PIP1–PIP2 interaction has been described as being necessary for the trafficking and relocalization of PIP1 proteins to the PM as a consequence of their physical interaction with some PIP2s [40,41]. A similar result was later obtained in epidermal cells of transgenic *Arabidopsis thaliana* roots [42]. The coexpression of PIP1–PIP2 was also demonstrated in *Fragaria ananassa* and *Beta vulgaris*, and it was shown to be responsible for an increased activity of PIP2 [39,43]. In addition, a different stoichiometry of AQPs isoforms was observed when they formed heterotetramers in the membrane of coinjected oocytes [43].

However, hetero-oligomerization in mammals was observed experimentally but with no clear function among the variants of monomers encoded by a similar gene, as in the case of AQP2 [13]. For AQP4, two polypeptide complexes formed with AQP, AQP4-M1, and AQP4-M2 were detected in the PM [44].

### 3.2. Diacidic and other Motifs

It has been shown that in yeast and animals [44], diacidic motifs or DIE (D/ExD/E sequences) in the cytosolic tails of transmembrane domains are involved in the export from the endoplasmic reticulum (ER) of different proteins. This diacidic signal evidenced that export from the ER occurred through a selective mechanism. However, the relationship of diacidic motifs with mammalian AQPs and their role in protein trafficking have not been studied in spite of the fact that these motifs have been found in the C-terminus tail of human AQP1 to AQP6, AQP9, and AQP10 and in the N-terminus of AQP8 [45]. It has also been observed that mutations in these motifs in AQP2 leads to disorders such as nephrogenic diabetes insipidus (NDI), with the retention of the aquaporins in the ER [45]. In a similar way, the importance of the N-terminus of AQP6 for its intracellular localization has been demonstrated in renal collecting ducts cells [46]. This trafficking regulation is critical for AQP6 function as its prolonged expression in the PM leads to cell death. In addition, a physical interaction of AQP6 with H+-ATPase in the vesicles of acid-secreting type-A intercalated cells of renal collecting ducts is able to regulate the vesicle pH. 

The importance of NPA (asparagine–proline–alanine) motifs for AQP trafficking has been demonstrated in AQP11, AQP12, and AQP4 [22,47,48], where variations of these motifs induced a lack of those AQPs in the plasma membrane.

In plants such as maize, diacidic motifs (Asp–Ile–Glu) at positions 4 to 6 in the N terminus of Zm-PIP2;4 and Zm-PIP2;5 were identified as playing an important role in export from the ER [49]. Similar results were found for *Arabidopsis thaliana* PIP2;1 [42]. However, the addition of a diacidic motif to Zm-PIP1;2 resulted in the retention of the protein in the ER. As this motif is not present in all plant AQPs, it may be involved exclusively in ER trafficking as other additional diacidic motifs (DAE) have also been identified in the N-terminus of Zm-PIP2;4 and Zm-PIP2;5 but they were not functional by themselves in ER trafficking. Even so, the involvement of a combination of both types of diacidic motif in proper AQPs export from the ER has not been ruled out [49].

### 3.3. Phosphorylation and Ubiquitination

Multiple post-translational modifications are essential for the regulation of the exocytosis of protein-containing vesicles. Thus, phosphorylation in the AQP2 C-terminus regulates protein trafficking, a process that is controlled by the pituitary antidiuretic hormone arginine vasopressin (AVP). Elevated levels of this hormone, in response to dehydration or hypernatremia, favor the binding of AVP to its receptor in the basolateral membrane, stimulating intracellular cAMP synthesis and subsequent Ser256 phosphorylation by PKA [35]. Other kinases, such as Golgi casein kinase (G-CK), may modulate Ser256-AQP2 phosphorylation prior to AQP2 translation from the Golgi to the vesicular post-Golgi compartment [50].

Although other residues, such as Thr269 (Ser269 in mice) and Ser264, are also phosphorylated in the presence of AVP [51,52], the only one identified so far as being involved in trafficking is Ser256, and the physiological significance of additional phosphorylations is not clear. Therefore, Ser256 phosphorylation is necessary for AQP2 maturation and its trafficking from the ER to the Golgi and from the Golgi to the post-Golgi compartment. Kamsteeg et al. [53] demonstrated by cRNA co-injection in oocytes, mimicking the phosphorylated and non-phosphorylated states of AQP2, that at least three monomers of the protein needed to be phosphorylated for steady-state PM localization. However, another study showed that the Ser256 phosphorylation of AQP2 was not enough to retain the protein at the PM [54], and the authors proposed that PKA-dependent phosphorylation of other proteins could also be part of the mechanism of AQP2 trafficking. In addition, a phosphorylation-independent mechanism has been shown for AQP2 recycling between intracellular storage compartments and the cell surface in renal cells [55].

In addition to protein kinases, phosphatases are also involved in AQP2 phosphorylation and trafficking, as Valenti et al. [56] demonstrated using okadaic acid—an inhibitor of serine/threonine phosphatases 1 and 2A (PP1 and PP2A). However, the mechanism by which these phosphatases exert their regulation is not clear, and an indirect effect of this inhibitor on actin filaments has been proposed [56]. In addition, in astrocytes, AQP4 translocation was shown to occur through a mechanism involving protein kinase A (PKA) activation and the influx of extracellular calcium and calmodulin, with Ser276 being the target residue of PKA for phosphorylation [57].

In plants, two Ser residues (Ser280 and Ser283) in *Arabidopsis thaliana* AtPIP2;1 have been described as phosphorylation targets. Phosphorylation of Ser283 is necessary for the trafficking of AtPIP2;1 from the ER to the PM [32], but mutations in Ser280 has no effect on AtPIP2;1 displacement under normal growth conditions. Under salt stress, the amount of protein in the PM decreased as a consequence of reduced migration from the ER and internalization of PIPs from the PM, with both phenomena being dependent on Ser283 phosphorylation. These results show the dynamic of PIP trafficking in plants in response to environmental stress such as salinity. 

Both mammalian and plant aquaporins have been shown to be ubiquitinated. Ubiquitination is one of the mechanisms involved in the endocytosis and lysosomal degradation of AQP2 in which the protein is polyubiquitinated for subsequent degradation in the proteasome [58]. It has been demonstrated that two hormones—prostaglandin E2 and dopamine—induce AQP2 internalization in a way that is independent of Ser256 phosphorylation [54,59]. Ubiquitin (Ub) binds covalently to a lysine residue at position 270 in AQP2. Similarly, in plants, the *Arabidopsis* aquaporin PIP2:1 has been found to show ubiquitination prior to its degradation [60]. In this study, the RING membrane-anchor 1 E3 Ub ligase, Rma1H1, played an important role in PIP2:1 trafficking from the ER to the PM, causing its inhibition in response to drought. 

## 4. Vesicles Containing Aquaporins and Communication between Cells

Communication between cells is an elementary function required for the proper development and maintenance of tissues and organs. For this, some classical mechanisms of interaction involving cell junctions, soluble factors, and contact adhesion may occur in the same cell or in others present in distant tissues [61]. However, the importance of other elements, such as extracellular vesicles (EVs), is becoming increasingly clear due to their wide variety of functions and their conservation at the phylogenetic scale [62]. 

EVs are defined as spheroids composed of cytosolic substance surrounded by a lipid bilayer with associated proteins, similar to the PM, which are released from cells into the surrounding environment [63,64]. Therefore, EVs serve as vehicles for the delivery of different signaling molecules, including proteins and lipids [65,66]. In particular, one of the most outstanding roles of EVs is the long-distance transport of RNA, which represents a complex mechanism of transcriptional regulation in both mammals and plants [67,68].

Despite the fact that EVs release was first observed in plants, the bulk of the knowledge in this field is based on work performed in mammals [69]. However, it is known that EVs are secreted from cells under both physiological (normal) conditions and in the presence of pathogens in both kingdoms [64]. In mammals, EVs are present in the majority of bodily fluids, such as saliva [70], cerebrospinal fluid [71], or breast milk [72]. The presence of exosomes in blood has been widely studied in the last few years, for instance, in the maturation of reticulocytes in erythrocytes [73,74,75]. Among the proteins involved in this maturation mechanism, it has been reported that a subpopulation of the water channel aquaporin-1 (AQP1) was selectively directed into multivesicular bodies and exosomes through a post-translational ubiquitination [76]. Exosomal secretion of AQP1 allowed the immature blood cells to deplete their pool in response to osmotic stress, suggesting a relevant role for AQP1 in this physiological process [77].

In addition, exosomes have also been shown to be involved in the transfer of functional proteins between cells. Specifically, in murine collecting duct cells from the kidney, it was reported that the transference of AQP2 to cells that did not previously express this water channel led to an increase in the water flow [78,79]. 

AQPs have also been related with an increase in the volume of vesicles before their secretion, which helps their fusion with the PM [80]. Specifically, the water channel AQP6 is involved in this process of swelling in the synaptic vesicles of neurons [81,82]. Vesicular acidification mediated by a H^+^-ATPase is a prerequisite for the correct gating of AQP6 and water transport. If an element involved in the vesicle swelling is impaired, it will probably affect the release of synaptic vesicles and therefore the neurotransmission process. Similarly, it has been reported that AQP1 is involved in the swelling process of zymogen granules present in the acinar cells of the exocrine pancreas, affecting cell function [83]. 

**Figure 1:**
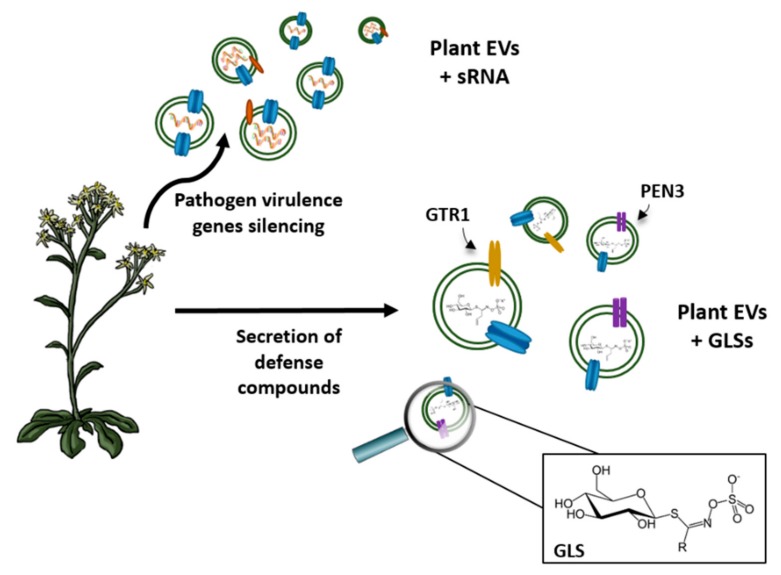
Against a pathogen attack, plants secrete extracellular vesicles (EVs) as a defense response. As seen above, plant EVs can contain small RNA (sRNA) that can silence virulence genes present in the pathogen. Another strategy is to secrete defense compounds, such as glucosinolates (GLSs), and include transport-related proteins, such as GTR1 (glucosinolate transporter-1) and PEN3 (an ATP-binding cassette transporter), in their membrane.

Plant cells also produce EVs; however, compared to mammals, little is known about their physiological role. The transport of important defense compounds produced under stress conditions is one of the main functions of these vehicles (Figure 1). Thus, in *Arabidopsis*, exosomes have been reported to be secreted in response to infection by the fungal pathogen *Botrytis cinerea*. In this case, the plant delivered host small RNAs (sRNA) in order to silence virulence-related genes in the fungal cells [84], which indicates that EVs may play an important role as cross-kingdom epigenetic regulators [85,86]. However, the presence of aquaporins in plant EVs needs further attention as these proteins are key elements involved in the response to abiotic and biotic stresses.

It has been demonstrated that EVs are involved in the long-distance transport of defense compounds; for example, different transporters of glucosinolates (GSLs) have been detected in plant EVs [87]. In this way, PEN3, which is supposed to unload these GSLs into the pathogen, and GTR1, involved in the movement of GSLs into and out of the phloem, have been found in EVs [87,88]. Therefore, EVs could serve as a secure storage site for these compounds, allowing their relocation in response to biotic infection [89]. The proteomic profile of plant EVs has unveiled the presence of phospholipases C and D, which contribute to the synthesis of phosphatidic acid [90]. This compound intervenes in the response to stress, activating the MAPK signaling pathway and the production of reactive oxygen species (ROS) [91]. Therefore, the authors suggested that plant EVs may act as signaling molecules in the receptor cells, activating the response to stress. However, a question related to plant EVs still remains unanswered: How are these large vesicles able to pass through the cell wall? Some hypotheses have been developed for other cells that possess a wall, such as mycoplasma or fungi, and also secrete EVs. One of these is based on the regulation of cell wall pore size, integrity, or thickness [92]. In bacteria, it has been shown that these properties can be modified by cell wall remodeling enzymes, such as glycerol phosphate lipoteichoic acid synthase, which loosen the cell wall to enable the release of EVs [93]. Another possibility is the direct association of EVs with these enzymes so that they are able to remodel the cell wall through their delivery [94]. 

Concerning the role of AQPs in plant EVs, no studies have demonstrated their presence in these vesicles. However, the fact that AQPs are located in EVs in plants should not be discarded as AQPs isoforms are abundant in membranes from which EVs can originate (TIPs in the vacuolar membrane and PIPs in the PM) [95,96]. Furthermore, the presence of AQPs in the lipid bilayer of PM-derived vesicles has been found to help the stabilization of GLSs [97]. As EVs have a role in the transport of these phytochemicals, as described previously, and require stabilization in order to travel long distances in the plant, these results could support the presence of AQPs in EVs. 

## 5. Industrial Application of Vesicles in Biomedicine

As mentioned previously, exosomes and vesicles are known to be key extracellular messengers in cell-to-cell communication. Therefore, their potential use in biomedicine has recently received attention [98]. Among the biomedical applications, these vesicles may be useful in diagnosis (as biomarkers) [99,100] and treatment (as vaccines) or as nanocarriers of drugs for the treatment of diseases [99,101,102].

Skog et al. [103] found that exosomes generated by cells reflected their state as they appeared under both normal physiological conditions and in the presence of disease or cellular damage but with a different composition and cargo. Therefore, the use of exosomes as biomarkers for disease diagnosis before the appearance of the first symptoms is starting to be helpful for several types of disease, such as neurodegenerative diseases [99], autoimmune diseases [104], cancer [105], and kidney diseases [106]. Exosomes are useful as biomarkers of cancer as well as for evaluating the results of surgery and detecting relapses in patients with cancer [107]. In fact, their contents, such as miRNAs, and their intrinsic proteins have been found to be related to the specific type of cancer, which represents an advance in the detection of the disease in early stages and in the development of personalized diagnoses [108]. 

**Figure 2:**
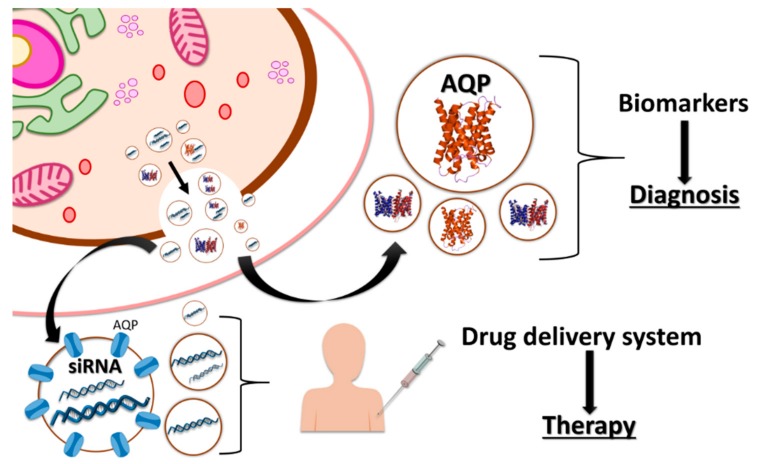
Exosomes containing aquaporins (AQPs) have been used to develop novel biomedical tools. As the abundance of AQPs in exosomes is modified under a specific disorder, they can be used as new biomarkers in the diagnosis of different diseases. Exosomes could also be used as drug delivery systems as they are able to cross biological barriers and guarantee the stability and bioavailability of drugs. One of the biological functions of exosomes is the delivery of RNA, based on which exosomes are used to transport siRNA used in therapies.

Among the proteins of exosomes, AQPs are the ones that have been studied the most [109]. As their main biological function is the regulation of water permeability of membranes—thus giving them an important role in cellular homeostasis—numerous studies have postulated them as key factors in the development of several human diseases [110]. For this reason, the link between AQPs and exosomes has prompted an interest in the development of novel biomedical tools (Figure 2). In fact, different studies have shown AQPs to be implicated in brain tumor pathogenesis [111,112]. AQP1 is important in tumor growth because it enhances cell growth and migration of cancer cells [113], while AQP4 has a crucial role in vasogenic oedema, a pathology with a considerable effect on the mortality related to brain tumors [111]. Thus, AQPs have a major function in brain tumor pathogenesis, and it has been postulated that brain cancer cells use exosomes to distribute AQPs between cells [112] in the same way as in other tissue systems, such as the kidney [113]. Therefore, exosomal AQPs in biological fluids could improve early diagnosis of these tumors as well as facilitate the monitoring of brain cancer progression. 

The identification and characterization of exosomes containing AQPs have given rise to a new path in the diagnosis and therapeutics of kidney diseases [114]. Among the AQPs proteins, AQP1 and AQP2 have been found in urinary exosomes, with a prospective use as biomarkers in several renal pathologies [115]. A common clinical syndrome with a high mortality is acute kidney injury (AKI). This disease is caused by ischemia-reperfusion (I/R) injury in many cases, which is mainly a consequence of the lack of early diagnostic markers for this pathology [116]. Sonoda et al. [114] showed that urinary exosomal AQP1 decreased in a rat model when a renal I/R injury appeared. In addition, they observed a decrease in exosomal AQPI in patients that had received a renal allograft transplant as renal I/R occurred during this process. Therefore, exosomal AQP1 can be used as a biomarker to detect renal I/R injury at the early onset and enable the monitoring of renal transplant patients and the prediction of post-transplant AKI caused by I/R. In a similar way to AKI, exosomal AQP1 has been reported as a potential biomarker for urinary tract obstruction [117] as this protein is less abundant in patients with this pathology. As a biomarker for this damage, AQP1 would be very useful, given existing clinical tests are not suitable for its prediction. 

In another assay carried out in a rat model, urinary exosomal AQP2 was described as a biomarker for early detection of gentamicin-induced renal injury, anticipating a future clinical application of this protein in diagnosis [118]. Gentamicin is an antibiotic used to treat many bacterial infections; however, several side-effects are associated with its use, such as nephrotoxicity. For this reason, a biomarker to detect gentamicin-induced renal injury would be interesting because traditional biomarkers of kidney injury, such as creatinine, do not permit early detection of this pathology. In their study, Abdeen et al. [118] found that urinary exosomal AQP2 increased on the first day after treatment with gentamicin; thus, exosomal AQP2 was able to show early renal damage caused by gentamicin. Besides, with a chronic gentamicin treatment, the urinary excretion of exosomal AQP2 was found to decrease, indicating again that this protein can be used to probe and check gentamicin-induced nephrotoxicity. Urinary exosomal AQP2 could also serve as a biomarker to predict or detect early pathologies associated with American cutaneous leishmaniasis (ACL), such as renal afflictions. In one study, urinary exosomal AQP2 levels were found to be lower in patients with ACL than in control patients, meaning that the reduced amount of AQP2 caused a urine-concentrating defect. In this case, exosomal AQP2 could be a suitable biomarker for this renal deficiency in patients with ACL [119].

In addition to their promise as biomarkers, exosomes could also be used as a drug delivery system. Exosomes have several advantages, including the fact that they are able to cross biological barriers [120,121], are biocompatible, can be autologous (patient-derived) [99], and can guarantee the stability and bioavailability of drugs [122]. Pocsfalvi et al. [109] detected AQPs in most mammalian exosomes, and these proteins are important elements in the maintenance of EVs stability [14], which is crucial for the application of these EVs in biomedicine. Therefore, AQPs present in mammalian exosomes could play an important role in achieving suitable EVs in terms of stability for use as drug delivery systems. As one of the biological functions of exosomes is the delivery of RNA, they can be used to transport the siRNA used in therapies. The first evidence of their suitability for this use was reported by Alvarez-Erviti [120]. The authors of this research proved that exogenous siRNA—loaded by electroporation into exosomes—triggered a specific mRNA, reducing its expression in the brain and leading to a 62% decrease in the accumulation of target protein in Alzheimer’s disease. 

Another application of great clinical interest is the use of this technology in the treatment of brain injuries. AQPs—especially AQP4, which is the most abundant AQP in the brain—have a key role in the formation of different cerebrovascular diseases [123] Therefore, AQP4 is a potential target for the treatment of this kind of injury or disease, with the objective of inhibiting AQP4 expression. Fukuda et al. (2013) [124] designed a siRNA targeting of AQP4 (siRNA-AQP4), and they reported that after siRNA treatment, there was a reduction in an oedema after a traumatic brain injury due to a decrease in AQP4 expression. This treatment had positive results, giving an important improvement in the patient after the lesions occurred. These types of drugs therefore represent a novel form of brain injuries therapy. In a similar manner, the delivery of the siRNA-AQP4 to the brain has been shown to produce a specific inhibition of the target protein without effects in other tissues or organs [120]. These results could lead to the development of an appropriate treatment for these pathologies using exosomes.

Therefore, exosomes have a great potential for use in the field of biomedicine, and as AQPs play an important role in many biological and pathological processes, the relationship between exosomes and AQPs with regard to their biotechnological applications in biomedicine is an interesting area to be exploited. 

## 6. Other Biotechnological Applications of Vesicles Containing Aquaporins

**Figure 3:**
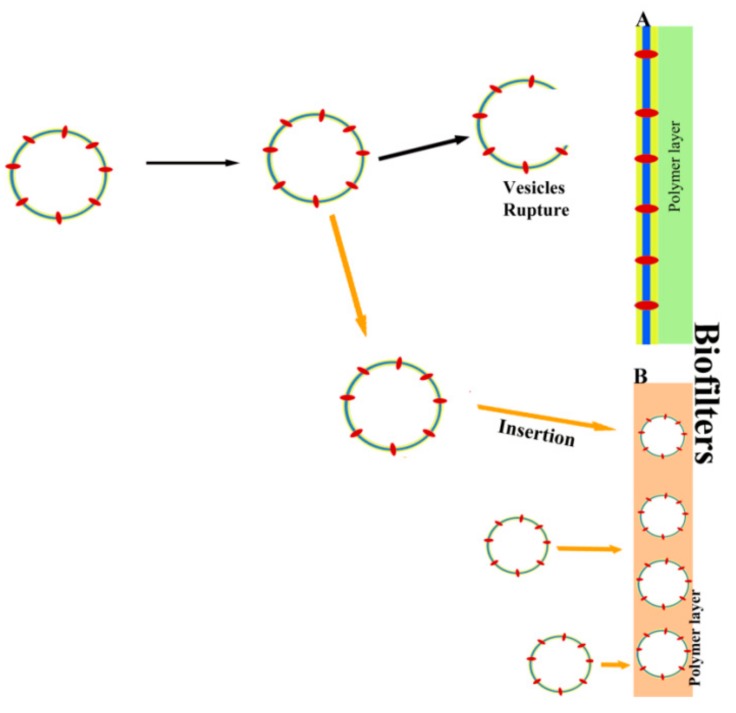
Schematic presentation of AQPs biofilter production. (**A**) AQPs lipid layer is made from vesicle rupture onto substrate layer by interaction between lipid and polymers. (**B**) Proteolipids vesicles are incorporated inside the polymers layer to make biofilter.

Additional biotechnological applications of vesicles containing AQPs have emerged in the area of biomaterials, such as the use of AQPs assemblies in membranes to create biofilters. Recent studies have focused on the search for new materials and strategies in order to increase the permeability and supply of membranes and thus achieve more efficient water desalination [125,126,127]. Ideally, a membrane with high permeability and strong salt rejection is needed to decrease energy expenditure. The use of ultrapermeable membranes has been shown to give an energy saving of up to 44% with the application of low pressure for seawater desalination [125]. Although thin-film composite membranes have been used in desalination processes, some studies have looked at ways to increase the efficiency of these membranes in terms of the material and structure. At the material level, one option explored was to incorporate new elements, such as AQPs, carbon nanotubes, and nanoporous grapheme, in order to increase membrane permeability [127]. Among these, AQPs represent the most innocuous solution due to their biological origin (Figure 3) [128].

AQPs are water transport channel proteins with a high specificity, and their potential to assemble in biomimetic membranes, which enhances their permeability and energy efficiency, has been demonstrated [128,129,130]. The AQP structure includes a pore with a diameter of 2.8 Å that confers on the protein the ability to allow water flux while rejecting solutes [131]. In addition, it has been reported that AQPs maintain their functionality after reconstitution in different membranes [132,133]. The main challenge is to create large vesicles bilayers containing AQPs [126,134]. In this sense, some studies have shown the viability of inserting AQPs from different biological samples of bovine and yeast origin to block copolymer membranes where the protein orientation is important for protein functionality and depends on the block copolymer symmetry. Thus, the possibility of incorporating AQPs into different block structures to design membranes for diverse technological uses has been studied [128,129]. However, the interaction between the polymers and the protein could be negative for the construction of membranes; this depends on their ratio and the composition of the block membrane. It is also necessary to prevent unfavorable electromagnetic interactions that could induce the formation of 2D crystals or a bad positioning of the proteins [134]. Thus, more studies have to be developed in this field to obtain the optimal ratios and lipid and polymer contents of the layers.

One of the main problems in the design of artificial membranes containing AQPs is the protein production, which is the main obstacle to industrial-scale production. In spite of their overexpression under biological conditions, AQPs have a complex structure with transmembrane regions, together with a low efficiency in different biological systems [135,136]. The most efficient process for AQPs generation has been suggested to be a “cell-free system” that yields AQPs in the presence of lipids or detergents, and the viability of the process has been demonstrated using diverse fusion vectors: pET28-AqpZ, pET32-AqpZ, and pET39-AqpZ [137,138]. In fact, this system could provide enough protein assembly in lipid layers with lower economic cost at an industrial scale. 

Several kinds of biomimetic membrane composed of AQP and phospholipid have been manufactured, and they have provided good results in desalination applications [127]. To construct biofilters, these AQP–lipid layer complexes may be integrated into polymer membranes. In this sense, there are different types of incorporation of AQPs into vesicles: AQP-incorporation supported in lipid layer membranes prepared by the vesicle rupture method and in membranes obtained from cell membranes [131,139]. To optimize the performance and function of membranes, different materials have been developed: supported lipid bilayer (SLB) and supported polymer membrane (SPM). Kaufman et al [113] indicated that in the case of SLBs, the interaction between the lipids and the substrate was key to the development of the membranes. Wang et al. [126] improved this design by a modification of the layers; based on the electrical properties of phospholipids, these authors proposed the layer-by-layer (LbL) assembly, where the support layer had a specific structure and composition so that the SLB and the LbL complex had opposing electrostatic charges. Wang et al [140] assembled an AQP through proteopolymersomes rupture on a modified polycarbonate tracked–etched substrate. Although this method could be easy to implement and offers advantages, such as good compatibility and fast transportation of water molecules, it also has disadvantages, such as easy rupture, bad scaling up, and expensive materials [110]. In order to avoid fragility of the AQP layer, Zhao et al [141] incorporated proteoliposomes into thin-film composite layers, where the final membrane could have an extensive area and be used at an industrial scale. Li et al. [142] showed that the combination of vesicles with other substances, such as polydopamine, increased the affinity with the matrix layer, thus augmenting its stability. In this sense, other studies have included recovered materials, such as magnetic nanoparticles [143], or amino acids, such as histidine, that binds to disulfide residues in the protein [140]. This binding avoids rupture in the manufacturing process and therefore prevents the vesicles from being peeled off by water flow. In addition, polymers such as polyethylenimine, polyamideimide, or polystryrene sulfonate protect the structure of AQPs against denaturation by chemical and biological agents. However, such systems have certain disadvantages, for example, the water molecules have to cross a double layer of lipids and the matrix layer introduces higher hydraulic resistance [136]. Thus, although commercial and highly efficient AQPs biofilters are available, more studies are necessary to improve the stability and functionality of these biomembranes. 

## 7. Conclusions

Plasma membrane vesicles containing AQPs have been studied in their biological environments, and their role in cell communication could be exploited for medicinal and commercial purposes. In animals, the AQP that has been studied the most is AQP2 in the kidney. AQP2 shares mechanisms of regulation with plant AQPs, such as the physical interaction with SNARE proteins that affects the pathway of AQP movement to the PM and therefore membrane water permeability. Another common form of regulation is phosphorylation, and distinct residues that can be phosphorylated have been identified. However, the involvement of others and of certain protein kinases still needs further investigation as this process will affect their biotechnological use. Vesicles containing AQPs are involved in cell-to-cell communication, with important signaling functions. Thus, vesicles incorporating AQPs could be useful as biomarkers of different AQPs-related disorders as the exosome proteomic profile may vary depending on the diagnosis. This role has not been explored for plant vesicles under different abiotic or biotic stresses, but this link between AQPs and exosomes in animals has been used to develop novel biomedical tools. In addition, exosomes could be used as drug delivery systems as they are able to cross biological barriers, are biocompatible, and can guarantee the stability and bioavailability of drugs. In this sense, their use as vehicles for siRNA is of great importance in different therapies. However, the transport and fusion of EVs through the membranes remain unexplored, and further studies are necessary in this direction. Finally, the role of AQPs as water channels make them optimal for use in biological membranes as biofilters. Although different techniques and materials have been developed in order to include AQPs vesicles in membranes, more studies are necessary to reduce costs and improve effectiveness.

Therefore, the overall scenario for the recently discovered role of vesicles containing aquaporins highlights the importance of aquaporins, not only in the physiology of organisms belonging to all kingdoms but also with respect to their further utilization in such vesicles in several fields.
